# Effects of Sleep Disturbances on Behavioral Problems in Preschool Children With Autism Spectrum Disorder

**DOI:** 10.3389/fpsyt.2020.559694

**Published:** 2021-02-09

**Authors:** Ying Wang, Jingjing Lin, Ying Zeng, Yanan Liu, Yamin Li, Kun Xia, Jingping Zhao, Yidong Shen, Jianjun Ou

**Affiliations:** ^1^Department of Psychiatry, National Clinical Research Center for Mental Disorders, China National Technology Institute on Mental Disorders, The Second Xiangya Hospital of Central South University, Changsha, China; ^2^Second Xiangya Hospital, Central South University, Changsha, China; ^3^State Key Laboratory of Medical Genetics, Central South University, Changsha, China

**Keywords:** autism spectrum disorder, sleep disturbance, behavioral problems, parasomnia, children's sleep habits questionnaire

## Abstract

**Study Objectives:** This study aims to identify the characteristic sleep disturbances that affect behavioral problems in children with autism spectrum disorder (ASD), providing a potential direction for sleep and behavioral intervention in ASD.

**Methods:** The data of 513 children with ASD and 246 typically developing (TD) children aged between 2 and 5 years old were collected. The behavior performance of preschool children was assessed using the Child Behavior Checklist for 1.5–5.0 years old. The Children's Sleep Habits Questionnaire (CSHQ) was used to measure the sleep status of the children, and the Social Responsiveness Scale was used to measure the severity of disorder. Linear regression analysis was performed to examine the effects of sleep disturbances on behavioral problems, and independent-sample *t*-test was performed to compare the mean of the samples.

**Results:** Compared to TD children, children with ASD had longer sleep onset delay and more night awakenings. The parasomnias score (a subscale of the CSHQ) was significantly associated with the internalizing (β = 0.113, *P* = 0.010), externalizing (β = 0.128, *P* = 0.006), and total problems (β = 0.143, *P* = 0.001) of children with ASD, while this association was not significant in TD children. “Bed wetting” and “restless and moves a lot” (two items in the CSHQ under parasomnia) significantly only affected the overall behavioral score in children with ASD (*P* < 0.05).

**Conclusion:** Parasomnias, especially bed wetting and restlessness, are specifically associated with the behavioral problems of children with ASD rather than TD children.

## Introduction

Autism spectrum disorder (ASD) is a neurodevelopmental disorder with core features of (1) deficits in social interaction and communication and (2) repetitive and stereotyped behaviors (1). This occurs in early childhood and seriously affects children and their families (2–4). Children with ASD are often afflicted with sleep disturbances and other conditions (5). In recent years, researchers have focused on the sleep problems of children with ASD, which have a higher prevalence (40–80%) when compared to typically developed (TD) children (25–40%) (6, 7). Furthermore, sleep problems in children with ASD are more likely to persist with age and contribute to more problems when compared to TD children (8, 9).

The relationship between sleep problems and ASD remains unclear. At present, there are three possible explanations: (1) the neural processes that control the sleep–wake cycle have changed due to the biological etiology of ASD, (2) sleep problems may be the clinical phenotype or core symptoms of ASD, and (3) sleep problems, including other mental states such as anxiety, serve as a comorbidity of ASD (10). The sleep characteristics of children with ASD commonly reported in the literature are greater sleep resistances, more sleep anxiety and night awakenings, and longer sleep latency as measured by subjective tools (9, 11–15). In addition, polysomnography studies have reported shorter sleep time, less rapid eye movement sleep, and awakening times in children with ASD (16). Parasomnia, such as enuresis and night terrors, have also been reported to be more frequent in children with ASD, but the conclusions have been inconsistent (9, 16).

Sleep plays an important role in the development and maturity of the brain and affects children's memory, attention, emotion, and behavior, especially in children with ASD (17). Sleep problems in children with ASD have a series of effects on their social function and performance, which not only aggravates autistic symptoms, such as social communication deficits, stereotypical behaviors, and interests (18–21), but also affects the behavior of children with ASD. Poor sleep is associated with a series of behavioral problems in children with ASD. The data from the Autism and Developmental Disabilities Monitoring Network and the Autism Speaks-Autism Treatment Network revealed that sleep problems are significantly associated with self-injurious behaviors (22). The previous study conducted by the investigators on children within 4–6 years old revealed that aggressive behaviors in children with ASD are significantly associated with sleep problems (23). It has also been reported that insomnia is associated with higher externalizing and total problems as measured by the Child Behavior Checklist (CBCL) (24).

Although a large number of studies have explored the association between sleep disturbance and behavioral problems in children with ASD, little has been determined on the effect of specific types of sleep problems (24, 25). Therefore, the investigators designed the present study to determine the association of specific sleep problems and behavioral problems in children with ASD. The present study has a very important implication. Defining these relationships can help develop specific sleep intervention strategies to improve behavioral problems, which can be an important direction in the intervention of children with ASD in the future. Indeed sleep problems are very common in mental disorders, and the intervention of sleep problems has been successfully used in anxiety disorder and major depression disorder (26).

## Methods

### Participants

Children with ASD were recruited from three special education schools and one public hospital, and TD children from nearby general kindergartens were recruited as controls. All children were within 2–5 years old. In order to ensure the homogeneity of the assessment, all subjects were called together at a combined time for the assessments. Furthermore, all children with ASD were evaluated by a senior child psychiatrist in order to confirm that they met the diagnostic criteria of autistic disorder or pervasive developmental disorder—not otherwise specified—in the Diagnostic and Statistical Manual for Mental Disorders, Fourth Edition, Text Revision. Subsequently, an independent child psychiatrist reconfirmed the diagnosis with a self-designed semi-structured diagnostic interview. The interview included reviewing the child's symptoms with the parents and observing the child's behavior during play with the child. Children with inconsistent diagnosis or Asperger syndrome were excluded from the present study. All children with serious physical diseases, such as congenital heart disease, renal insufficiency, and genetic diseases such as Down syndrome, were excluded. Furthermore, TD children who were reported to have serious learning, behavioral, emotional, or social problems by parents and teachers or have a family history of ASD or other mental illness were also excluded. None of these children had taken any psychiatric-related drugs. The Social Response Scale (SRS) was used to measure autistic traits in the general population. The diagnostic sensitivity is the best, with a total raw cutoff score of 56.5 among the Chinese mainland population (27). To achieve the best diagnostic sensitivity, only ASD with a total score of SRS >56.5 and TD with a total score of SRS <56.5 were included. The R 3.6.2 software and “pwr” package were used to estimate the sample size. When the correlation coefficient was set as 0.2, the power was set as 0.8, and the significant level was set as 0.05. The required sample size of each group was 193.

The present study was approved by the Ethics Committee of Second Xiangya Hospital and was conducted from January 2016 to April 2019. The guardians of all participants provided a written informed consent prior to enrollment.

### Measures

The Child Behavior Checklist for 1.5–5.0 years old is a scale widely used to assess behavioral and emotional problems in preschoolers. (28). It contains 99 items, in which parents evaluate the behavior of children in the past 2 months using a scale from 0 (not true) to 2 (very true or often true). This scale includes eight clinical syndrome subscales (emotionally reactive, anxious/depressed, somatic complaints, withdrawn, sleep problems, attention problems, aggressive behavior, and stress) and three broad-spectrum scales (internalizing problems, externalizing problems, and total problems). All rough scale scores were converted into T scores according to the norm of the Chinese population in this study.

The Children's Sleep Habits Questionnaire (CSHQ) is designed for parents and is used to assess sleep problems in school-age children by reviewing their sleep performance within a week. (29). Several studies have found this to be clinically useful for assessing the sleep status of children within 2–5 years old (14, 30), including children with ASD and other neurodevelopmental disorders. The questionnaire includes 33 items, ranging from “very few” to “usual,” with a score of 1 to 3. Sleep problems are assessed on eight subscales: (1) bedtime resistance, (2) sleep onset delay, (3) sleep duration, (4) sleep anxiety, (5) night awakening, (6) parasomnias, (7) sleep disordered breathing, and (8) daytime drowsiness. Higher total and subscale scores mean more serious sleep problems.

The social response scale (SRS) can be used to compare autism-like traits across clinical and non-clinical populations. It focuses on the behavior of children over the past 6 months. This scale applies to all participants and is not affected by intelligence, age, race, or the level of education of the interviewers (31). The questionnaire consists of 65 items and is meant to be completed by parents, teachers, or other regular caregivers. The SRS has been translated into many languages, but there is no norm for T-score conversion in the Chinese version. Hence, the raw score was used for the present study.

### Statistical Analysis

Chi-square test was used to compare the gender of the two groups. Independent-sample *t*-test was used to compare the age of the children, the age of their parents, the total score of the SRS, the three broad-spectrum scale score of the CBCL, the total score of the CSHQ, and the scores of the different sleep domains. The children's age, gender, and SRS total score were taken as control variables, and eight sleep disturbance domain variables were all entered into the linear regression model to test the effect of specific sleep problems on the different dimensions of CBCL (25). The linear regression model was also used to determine the effect of the CSHQ total score and each item of parasomnias and sleep disordered breathing on the different dimensions of CBCL. None of the missing data were included in the regression analysis. The severity of behavioral problems and symptoms in children, with or without bed wetting, restless, and snores, were compared using independent-sample *t*-test. The level of significance was defined as *P* < 0.05. All statistical analyses were carried out using the SPSS 23.0 software.

## Results

### Characteristics of the Participants

In the present study, 513 children with ASD and 246 TD children were recruited. However, after excluding the missing values and screening according to the criteria for the SRS cut point, merely 474 children with ASD and 193 TD children remained and were included for the next analysis. There were significant differences in gender composition (χ^2^ = 65.446, *p* < 0.001) and father's age (*t* = −2.244, *p* = 0.025) between children with ASD and TD children. Boys accounted for a higher proportion, and the fathers were older in the ASD group. There was no significant difference in the age of children and mothers between the two groups. The total score for the SRS was significantly higher in the ASD group when compared to the TD group, and similar results were found for all dimensions of CBCL (all *P* < 0.001). There was no significant difference in the total score for the CSHQ between the two groups (*t* = were compared using independent 0.227, *P* = 0.821). Compared with TD children, children with ASD had longer sleep latency (*t* = −4.255, *P* < 0.001) and more awakening times (*t* = −2.155, *P* = 0.032) at night, while TD children had higher scores of sleep anxiety (*t* = 4.694, *P* = 0.000) when compared to children with ASD. However, there was no significant difference in the other sleep dimensions between the two groups (all *P* < 0.05) (Table 1).

**Table 1:** Participant demographics and clinical characteristics.

	**ASD(*N* = 474)**	**TD(*N* = 193)**	***t/χ^2^***	***P***	
Sex(M/F)	402/72	107/86	65.446	<0.001	***
Age(year)	3.73 (1.01)	3.71 (0.86)	−0.233	0.816	
Father's age(year)	34.59 (4.61)	33.70 (4.38)	−2.244	0.025	*
Mother's age(year)	32.89 (4.06)	32.52 (3.81)	−1.057	0.291	
SRS Total score	93.26 (22.23)	35.51 (10.87)	−42.922	<0.001	***
CBCL/1.5-5					
Internalizing problems	59.68 (9.29)	43.03 (13.16)	−16.024	<0.001	***
Externalizing problems	51.95 (8.67)	39.50 (11.76)	−13.301	<0.001	***
Total problems	56.70 (10.05)	40.77 (12.72)	−15.528	<0.001	***
CSHQ total score	46.48 (6.25)	46.36 (6.14)	−0.227	0.821	
Bedtime resistance	11.71 (2.16)	11.40 (2.20)	−1.673	0.095	
Sleep onset delay	1.88 (0.75)	1.62 (0.70)	−4.255	<0.001	***
Sleep duration	4.86 (1.72)	4.78 (1.76)	−0.536	0.592	
Sleep anxiety	6.41 (1.72)	7.17 (1.95)	4.694	<0.001	***
Night awakening	3.51 (1.08)	3.35 (0.75)	−2.155	0.032	*
Parasomnias	7.81 (1.72)	8.00 (1.84)	1.295	0.196	
Sleep disordered breathing	3.35 (0.95)	3.24 (0.70)	−1.706	0.089	
Daytime sleepiness	11.61 (2.70)	11.43 (2.58)	−0.781	0.435	

*0.01 < p ≤ 0.05; ***p ≤ 0.001.

*SRS, social responsiveness scale; CBCL/1.5-5, child behavior checklist version for preschool children, CSHQ, children's sleep habits questionnaire. Results are presented with the mean and the standard deviation*.

### Linear Regression Analysis of Sleep Disturbances on Behavioral Problems

After controlling for the age, gender and SRS total score of children, the linear regression revealed that the total score of the CSHQ had a significant effect on the behavior problems of children in both groups, including the eight-clinical-syndromes scale and the three-broad-spectrum scale (all, *P* < 0.01; Table 2 and Supplementary Table 1). Parasomnias were significantly associated with the internalizing (β = 0.114, *P* = 0.010), externalizing (β = 0.130, *P* = 0.006), and total (β = 0.143, *P* = 0.001) problems of children with ASD (Table 2) as well as the five-clinical-syndromes subscale (Supplementary Table 1), while this effect was not significant in TD children (all *P* > 0.05). Sleep disordered breathing was only associated with behavioral problems in TD children, involving the eight-clinical-syndromes scale and the three-broad-spectrum scale (all, *P* < 0.05; Table 2 and Supplementary Table 1). As for the other sleep dimensions, sleep duration had a significant effect on externalizing problems (β = 0.097, *P* = 0.026) of children with ASD, including attention and aggressive behavior problems, while this was mainly associated with the internalizing problems (β = 0.175, *P* = 0.031) of TD children. In addition, sleep duration was significantly correlated with sleep problems and overall behavioral problems in the two groups (all *P* < 0.05). Sleep anxiety was associated with the internalizing problems (β = 0.104, *P* = 0.025) of children with ASD, including anxiousness/depression and stress, while this was associated with the externalizing (β = 0.216, *P* = 0.024) and total (β = 0.234, *P* = 0.013) problems in TD children. Night awakening only affected the internalizing (β = −0.220, *P* = 0.005) and total problems (β = −0.215, *P* = 0.005) in TD children. Daytime sleepiness was only associated with internalizing problems (β = 0.217, *P* = 0.031), which was embodied in being emotionally reactive and stress (all *P* < 0.05) in children with ASD (Table 2 and Supplementary Table 1).

**Table 2:** Linear regression analysis of sleep disturbances on children behavior problems.

	**Internalizing problems**						**Externalizing problems**						**Total problems**					
	**ASD**			**TD**			**ASD**			**TD**			**ASD**			**TD**		
	**β**	***P***		**β**	***P***		**β**	***P***		**β**	***P***		**β**	***P***		**β**	***P***	
CSHQ Total Score^a^	0.215	<0.001	***	0.35	<0.001	***	0.226	<0.001	***	0.372	<0.001	***	0.275	<0.001	***	0.399	<0.001	***
Bedtime resistance^b^	0.013	0.776		0.046	0.640		0.034	0.454		−0.010	0.916		0.024	0.598		0.013	0.897	
Sleep onset delay^b^	0.065	0.113		−0.026	0.749		0.062	0.182		−0.010	0.901		0.075	0.055		−0.008	0.923	
Sleep duration^b^	0.035	0.398		0.175	0.031	*	0.097	0.026	*	0.156	0.056		0.096	0.017	*	0.187	0.019	*
Sleep anxiety^b^	0.104	0.025	*	0.216	0.024	*	0.006	0.906		0.191	0.047		0.074	0.096		0.234	0.013	*
Night awakening^b^	0.003	0.951		−0.220	0.005	**	−0.006	0.715		−0.162	0.038		0.010	0.808		−0.215	0.005	**
Parasomnias^b^	0.114	0.010	**	0.090	0.310		0.130	0.006	**	0.149	0.097		0.143	0.001	***	0.115	0.188	
Sleep disordered breathing^b^	0.003	0.935		0.217	0.013	*	0.033	0.411		0.223	0.012	*	0.018	0.637		0.225	0.008	**
Daytime drowsiness^b^	0.080	0.037	*	0.059	0.447		0.055	0.274		0.092	0.242		0.072	0.053		0.093	0.226	

*0.01 < p ≤ 0.05; **0.001 < p ≤ 0.01; ***p ≤ 0.001.

a *Age of children, sex and SRS total score were taken as predictive variables, and CSHQ total score was taken as independent variable*.

b *Age of children, sex and SRS total score were taken as predictive variables, all CSHQ subscales were included in the regression equation*.

*Internalizing problems, externalizing problems, and total problems are three important overall subscales of the CBCL*.

### The Relationship Between Specific Parasomnia Items and Behavioral Problems of Children

A previous analysis revealed that parasomnias are specifically associated with the behavioral problems of children with ASD. In order to identify the specific sleep indicators associated with behavioral problems in children with ASD, different parasomnia items were included as predictor variables to determine the effects on children's behavioral problems. Children's age and the SRS total score were taken as covariates. Table 3 revealed that “wets the bed” was associated with the internalizing (β = 0.112, *P* = 0.007), externalizing (β = 0.100, *P* = 0.023), and overall behavioral (β = 0.120, *P* = 0.003) problems in children with ASD, but not in TD children. Supplementary Table 2 presents this effect, referring to the four clinical syndromes: somatic complaints, being withdrawn, sleep problems, and aggressive behavior. “Restless and moves a lot” had an effect on the externalizing (β = 0.087, *P* = 0.046) and overall behavior (β = 0.089, *P* = 0.025) of children with ASD. However, this had no effect on TD children (Table 3 and Supplementary Table 2). Supplementary Table 2 shows this effect, referring to the three clinical syndromes: somatic complaints, sleep problems, and attention problems. “Awakens screaming” had an effect on the total problems (β = 0.097, *P* = 0.028) of children with ASD, including somatic complaints and sleep problems. “Sleepwalks” and “grinds teeth” was associated with the somatic complaints (all *P* < 0.05) of children with ASD, while “nightmares” were associated with the emotionally reactive problems (β = 0.114, *P* = 0.019), sleep problems (β = 0.126, *P* < 0.001), aggressive behavior (β = 0.160, *P* = 0.001), and stress problems (β = 0.096, *P* = 0.043) of children with ASD (Supplementary Table 2).

**Table 3:** Linear regression analysis of parasomnias items on children behavior problems^a^.

**Parasomnias items of CSHQ**	**Internalizing problems**					**Externalizing problems**					**Total problems**				
	**ASD**			**TD**		**ASD**			**TD**		**ASD**			**TD**	
	**β**	***P***		**β**	***P***	**β**	***P***		**β**	***P***	**β**	***P***		**β**	***P***
Wets the bed	0.112	0.007	**	−0.029	0.740	0.100	0.023	*	0.000	0.999	0.120	0.003	**	−0.011	0.896
Talks during sleep	0.020	0.609		0.071	0.446	0.006	0.883		0.135	0.144	0.036	0.357		0.112	0.229
Restless and moves a lot	0.076	0.063		0.021	0.807	0.087	0.046	*	0.046	0.589	0.089	0.025	*	0.028	0.741
Sleepwalks	−0.009	0.813		−0.036	0.680	−0.055	0.192		−0.018	0.835	−0.031	0.415		−0.028	0.749
Grinds teeth	0.029	0.458		0.016	0.850	0.075	0.078		0.042	0.627	0.050	0.201		0.010	0.909
Awakens screaming	0.072	0.109		0.128	0.191	0.033	0.490		0.146	0.133	0.097	0.028	*	0.143	0.142
Nightmares	0.008	0.858		0.163	0.106	0.076	0.109		0.078	0.437	0.042	0.335		0.125	0.215

*0.01 < p ≤ 0.05; **0.001 < p ≤ 0.01.

a *Age of children, sex and SRS total score were taken as predictive variables, all parasomnias items were included in the regression equation*.

*Internalizing problems, externalizing problems, and total problems are three important overall subscales of the CBCL*.

**Figure 1:**
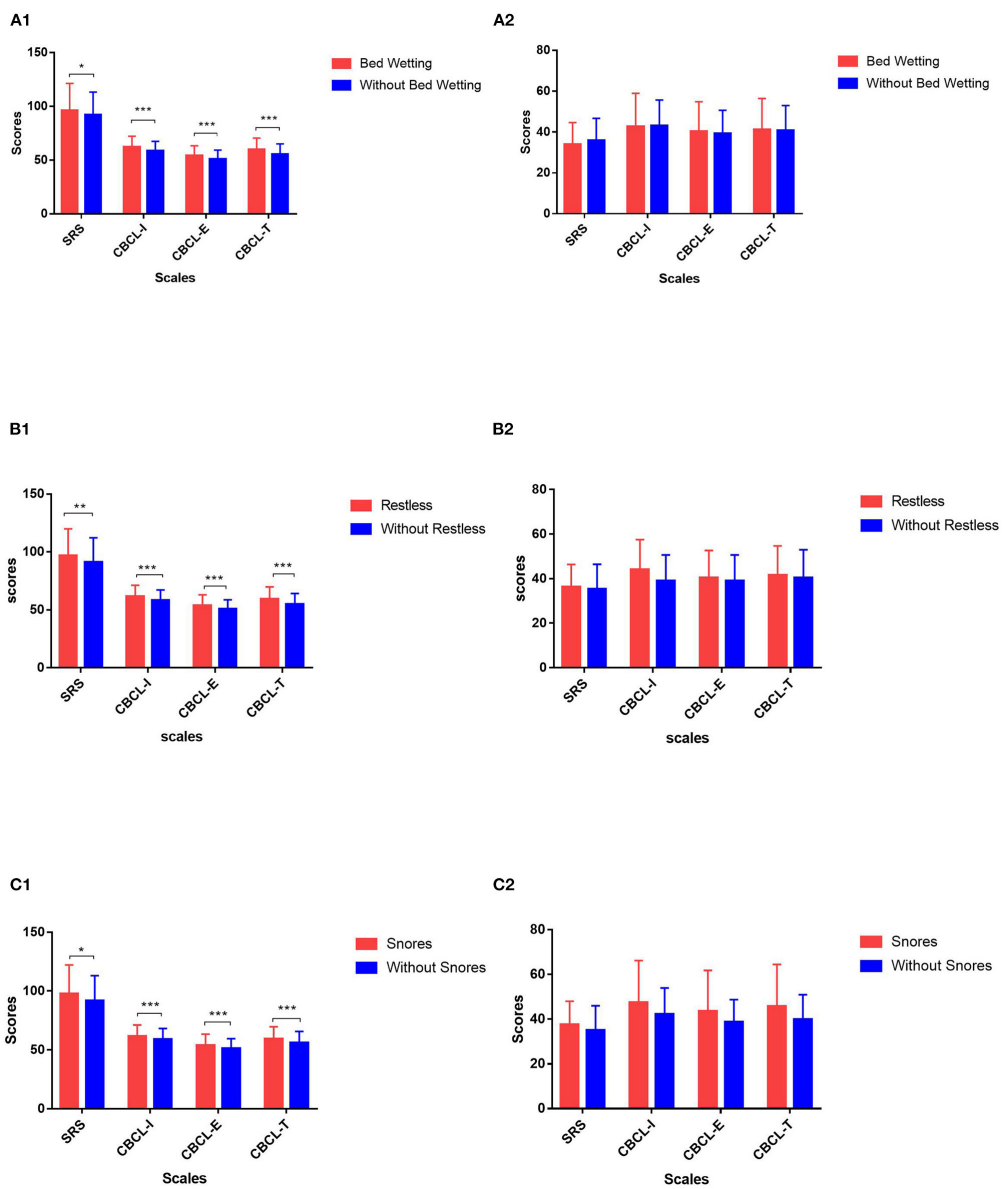
Comparison of behavior and symptom overall scores of children with or without specific sleep problems in different groups. **(A)** 1: Comparison of Social Responsiveness Scale (SRS) and Child Behavior Checklist (CBCL) scores in children with autism spectrum disorder (ASD) with or without bed wetting. 2: Comparison of SRS and CBCL scores in typically developing (TD) children with or without bed wetting. **(B)** 1: Comparison of SRS and CBCL scores in children with ASD with or without restlessness during sleep. 2: Comparison of SRS and CBCL scores in TD children with or without restlessness during sleep. **(C)** 1: Comparison of SRS and CBCL scores in children with ASD with or without snoring loudly. 2: Comparison of SRS and CBCL scores in TD children with or without snoring loudly. SRS, Social Responsiveness Scale; CBCL- I, internalizing problem score of CBCL; CBCL- E, externalizing problem score of CBCL; CBCL- T, total problem score of CBCL. *0.01 < *p* ≤ 0.05; **0.001 < *p* ≤ 0.01; ***p ≤ 0.001.

Among the seven items for parasomnias, “wets the bed” and “restless and moves a lot” had higher probabilities of occurrence in children with ASD (all *P* < 0.05), and the incidence for girls was higher than that for boys (Supplementary Table 3). According to the situation of bed wetting and restlessness, the scores for the SRS and CBCL in the different subgroups were compared. The “wets the bed” item with a score of “1” was considered to be without bed wetting, while the other scores were regarded as having bed wetting, and this was similar to “restless and moves a lot.” Figure 1 shows that, among children with ASD, the scores of the SRS and CBCL in children with bed wetting and restlessness were higher than those without these. However, there was no significant difference in SRS and CBCL scores between TD children with or without bed wetting and restlessness.

### The Relationship Between Specific Sleep Disordered Breathing Items and Behavioral Problems of Children

It was also found that sleep disordered breathing was specifically associated with the behavioral problems of TD children in the previous analysis. In order to further explore the effect of sleep disordered breathing on the behavior of children in these two groups, different sleep disordered breathing items were included as predictor variables to determine the effects on children's behavioral problems. Children's age, gender, and the SRS total score were taken as the covariates. Table 4 and Supplementary Table 4 revealed that “snores loudly” affected the total problems of TD children (β = 0.083, *P* = 0.042), including somatic complaints and attention problems.

**Table 4:** Linear regression analysis of sleep disordered breathing items on children behavioral problems^a^.

**Sleep Disordered Breathing items of CSHQ**	**Internalizing problems**				**Externalizing problems**				**Total problems**			
	**ASD**		**TD**		**ASD**		**TD**		**ASD**		**TD**	
	**β**	***P***	**β**	***P***	**β**	***P***	**β**	***P***	**β**	***P***	**β**	***P***
Snores loudly	0.050	0.231	0.140	0.113	0.069	0.116	0.139	0.111	0.083	0.042 *	0.162	0.065
Stops breathing	0.026	0.606	0.139	0.197	0.006	0.916	0.169	0.114	0.008	0.874	0.125	0.246
Snorts and gaps	0.002	0.970	0.073	0.490	0.031	0.559	0.089	0.397	0.024	0.624	0.093	0.376

*p < 0.05.

a *Age of children, sex and SRS total score were taken as predictive variables. All sleep disordered breathing items were included in the regression equation*.

*Internalizing problems, externalizing problems, and total problems are three important overall subscales of the CBCL*.

Supplementary Table 3 shows that, among the three items of sleep disordered breathing, the incidence of “snores loudly” was the highest, while the incidence of “stops breathing” and “snorts and gasps” were low in both groups. Among the children with sleep breathing disorders, 81.6% were combined with parasomnias at the same time. According to the situation of snores, the scores of the SRS and CBCL in the different subgroups were compared. The “snores loudly” item with a score of “1” was considered to be without snores, while other scores were regarded as having snores. Figure 1 shows that, among children with ASD, the scores of the SRS and CBCL in children with and without snoring loudly were higher than those without these. However, there was no significant difference in TD children.

## Discussion

In the present study, the effects of sleep disturbances on behavioral problems were examined between ASD and TD children based on the parental feedback data. A significant difference was found in the effect of parasomnias on behavioral problems in children with ASD, but not in TD children. Among the seven items of parasomnias, “wets the bed” and “restless and moves a lot” had a relatively higher incidence, and these were only associated with the behavioral problems of children with ASD but had no significant effect on the behavioral problems of TD children. The grouping analysis also suggested that, in the ASD group, children with bed wetting and restlessness were more seriously ill and had more behavioral problems, while there was no such effect in TD children.

### Characteristics of Sleep Disturbances in Pre-school Children With ASD

In the present study, it was found that there were longer sleep latency and more night awakenings in children with ASD, which was consistent with previous studies (9, 14, 15). However, the sleep anxiety reported in the present study was higher in TD children than in children with ASD, while studies in Western countries reported more sleep anxiety in children with ASD. However, studies based on Chinese samples were consistent with the results of this study, suggesting that this difference might be caused by nationality or cultural differences (15). Chinese parents with children with ASD may lack the knowledge to identify their children's sleep anxiety for their expression defects. There were no significant differences in other sleep dimensions and overall sleep problems in the present study, and these results were not consistent with those of other studies, which may be correlated to the children's age, individual heterogeneity, and cultural differences. Children with ASD have different degrees of prominent sleep problems in different age stages (32). The present study was limited to 2- to 5-year-old children, which offers an important reference value for the sleep characteristics of preschoolers.

### The Relationship Between Sleep Disturbances and Behavioral Problems

A large number of studies have revealed that children with ASD with poor sleep had more behavioral problems (22, 23). The advantage of the present study is that the investigators studied the effects of the different sleep problems reported by parents as measured by the CSHQ on the different behavioral problems measured by the CBCL. Through linear regression analysis, the present study revealed that parasomnias were specifically associated with the internalizing, externalizing, and overall behavior problems in children with ASD, but not in TD children. A previous study also suggested that sleep resistance, night awakenings, and total sleep problems could predict the daytime behavior of ASD and TD children (25). These studies also attempted to explore the effects of sleep problems on daytime behavior but only chose daytime sleep as the main independent variable, and the results were negative (25). In the present study, the investigators not only found that parasomnia is the characteristic sleep index that affects the daytime behavior of children with ASD but also analyzed different items. It was found that “wets the bed” and “restless and moves a lot” were associated with the behaviors of children with ASD, but not TD children, and that these problems had relatively higher incidences in children with ASD. Autistic children with bed wetting and restlessness were accompanied through more behavioral problems, and their clinical symptoms were more serious. However, for TD children with or without bed wetting and restlessness, there was no significant difference in behavioral problems. This further confirms that bed wetting and restlessness are characteristic sleep disturbances that affect the behavior problems of children with ASD.

The linear regression analysis revealed that sleep breathing disorders were mainly associated with the behavioral problems of TD children, but not children with ASD. The further item analysis revealed that there was no item that only affects the behavioral problems of TD children. The linear regression analysis revealed that “snores loudly” had an effect on certain behavior problems of children in both groups. However, when compared with children with ASD, children who snored had more behavior problems than those who did not snore, but no such difference was found in TD children. In the present study, the incidence of “stops breathing” and “snorts and gasps”—two items of sleep breathing disorder—was relatively low, so the effect of these two items on the behavioral problems of pre-school children remains to be further verified after expanding the sample size. The linear regression analysis revealed that sleep breathing disorders only affect the behavioral problems of TD children, while the further analysis revealed different results. In the present study, children who snore were often associated with parasomnias, which may be the reason why this effect was masked in the regression analysis.

### Potential Directions for Future Interventions

According to the bidirectional theory, the effects of sleep disturbance and behavioral problems are mutual (5). In addition, sleep may indirectly affect the behavior of children with ASD by affecting cognitive function. Sleep problems can reduce flexibility, delay language acquisition, and hinder the development of skills, thereby increasing the risk of externalizing behaviors (33). Furthermore, this also interacts with internalizing behaviors (5). Sleep disturbance and behavioral problems are both prominent in children with ASD and interact with each other. Interventions should be taken to break this vicious circle. Compared with the intervention of behavioral problems, it is more convenient to correct sleep problems, and the results could easily be observed. The present study determined the characteristic sleep problems associated with the behavior of children with ASD, which may provide a potential direction for sleep intervention in the future.

## Limitations

There were still some limitations in the present study. First, there was an imbalance between the case group and the control group, and there was a significant gender difference between these two groups, which was due to the gender difference in the incidence of ASD. In order to reduce the influence of gender, the investigators used gender as the control variable in the present analysis. Second, the present study used the questionnaire reported by parents to measure the sleep problems of children, which lack objective measurement indicators. Furthermore, there may be omissions on important information due to parent's negligence. Third, the number of controls was <50% of the cases, which affected the power of the study. Finally, the CSHQ used in the present study has good reliability and validity for children within 4–10 years old, but there is no consistent study on its application in children under 4 years old. There was a large number of children within 2–4 years old in the present study. Hence, the CSHQ may not fully reflect the sleep problems of these children.

## Conclusion

In summary, the investigators determined the effects of sleep problems on behavioral performance in children with ASD who were within 2–5 years old. It was found that parasomnias, especially bed wetting and restlessness, were specifically associated with the behavioral problems of children with ASD when compared to TD children. This implies that intervention on parasomnias may be an important direction for improving the behavioral problems of children with ASD.

## Data Availability Statement

The original contributions presented in the study are included in the article/Supplementary Material, further inquiries can be directed to the corresponding author/s.

## Ethics Statement

The studies involving human participants were reviewed and approved by Ethics Committee of the second Xiangya Hospital. Written informed consent to participate in this study was provided by the participants' legal guardian/next of kin.

## Author Contributions

YW and JL made a statistical analysis and wrote the manuscript, as co-first authors. JO and YS jointly designed the experimental scheme and supervised the implementation of the experiment, as the common communication authors. YZ and YanL participated in the data collection. YamL, KX, and JZ participated in the improvement and revision of the experimental scheme. All authors contributed to the article and approved the submitted version.

## Conflict of Interest

The authors declare that the research was conducted in the absence of any commercial or financial relationships that could be construed as a potential conflict of interest.
