# Supplementary material for: Effects of Sleep Disturbances on Behavioral Problems in Preschool Children With Autism Spectrum Disorder
Source: Front Psychiatry. 2021 Feb 9;11:559694. doi: 10.3389/fpsyt.2020.559694 (PMC7900493; doi:10.3389/fpsyt.2020.559694)
Supplement: Supplementary file 1 [file Data_Sheet_1.docx]

Table S1. Linear regression analysis of sleep disturbances on different children behavior problems

|  | ASD | | |  | TD | | |
| --- | --- | --- | --- | --- | --- | --- | --- |
|  | β | p |  |  | β | p |  |
| **Emotionally reactive** |  |  |  |  |  |  |  |
| CSHQ Total Score ^a^ | 0.208 | <0.001 | ******* |  | 0.310 | <0.001 | ******* |
| bedtime resistance ^b^ | -0.041 | 0.444 |  |  | 0.029 | 0.787 |  |
| sleep onset delay ^b^ | 0.041 | 0.360 |  |  | -0.050 | 0.570 |  |
| sleep duration ^b^ | 0.052 | 0.256 |  |  | 0.147 | 0.090 |  |
| sleep anxiety ^b^ | 0.033 | 0.516 |  |  | 0.113 | 0.268 |  |
| night awakening ^b^ | 0.038 | 0.403 |  |  | -0.016 | 0.847 |  |
| Parasomnias ^b^ | 0.132 | 0.007 | ****** |  | 0.093 | 0.328 |  |
| sleep disordered breathing ^b^ | 0.030 | 0.502 |  |  | 0.218 | 0.020 | ***** |
| daytime sleepiness ^b^ | 0.100 | 0.020 | ***** |  | 0.039 | 0.640 |  |
| **Anxious/depressed** |  |  |  |  |  |  |  |
| CSHQ Total Score ^a^ | 0.216 | <0.001 | ******* |  | 0.355 | <0.001 | ******* |
| bedtime resistance ^b^ | -0.036 | 0.508 |  |  | 0.044 | 0.666 |  |
| sleep onset delay ^b^ | 0.043 | 0.345 |  |  | -0.033 | 0.698 |  |
| sleep duration ^b^ | 0.08 | 0.089 |  |  | 0.098 | 0.238 |  |
| sleep anxiety ^b^ | 0.132 | 0.012 | ***** |  | 0.166 | 0.091 |  |
| night awakening ^b^ | -0.014 | 0.757 |  |  | -0.070 | 0.373 |  |
| Parasomnias ^b^ | 0.121 | 0.015 | ***** |  | 0.167 | 0.068 |  |
| sleep disordered breathing ^b^ | 0.059 | 0.199 |  |  | 0.246 | 0.006 | ****** |
| daytime sleepiness ^b^ | 0.068 | 0.119 |  |  | 0.027 | 0.741 |  |
| **Somatic complaints** |  |  |  |  |  |  |  |
| CSHQ Total Score ^a^ | 0.206 | <0.001 | ******* |  | 0.338 | <0.001 | ******* |
| bedtime resistance ^b^ | 0.000 | 0.995 |  |  | 0.051 | 0.617 |  |
| sleep onset delay ^b^ | 0.026 | 0.584 |  |  | 0.020 | 0.812 |  |
| sleep duration ^b^ | 0.055 | 0.254 |  |  | 0.111 | 0.183 |  |
| sleep anxiety ^b^ | 0.007 | 0.902 |  |  | 0.079 | 0.422 |  |
| night awakening ^b^ | -0.028 | 0.559 |  |  | -0.072 | 0.364 |  |
| Parasomnias ^b^ | 0.258 | <0.001 | ******* |  | 0.092 | 0.313 |  |
| sleep disordered breathing ^b^ | -0.019 | 0.691 |  |  | 0.339 | <0.001 | ******* |
| daytime sleepiness ^b^ | 0.053 | 0.236 |  |  | 0.013 | 0.870 |  |
| **Withdrawn** |  |  |  |  |  |  |  |
| CSHQ Total Score ^a^ | 0.110 | 0.003 | ****** |  | 0.324 | <0.001 | ******* |
| bedtime resistance ^b^ | 0.064 | 0.172 |  |  | 0.029 | 0.786 |  |
| sleep onset delay ^b^ | 0.070 | 0.080 |  |  | 0.005 | 0.942 |  |
| sleep duration ^b^ | 0.043 | 0.282 |  |  | 0.126 | 0.128 |  |
| sleep anxiety ^b^ | 0.029 | 0.514 |  |  | 0.130 | 0.193 |  |
| night awakening ^b^ | -0.015 | 0.706 |  |  | -0.067 | 0.401 |  |
| Parasomnias ^b^ | 0.013 | 0.754 |  |  | 0.141 | 0.13 |  |
| sleep disordered breathing ^b^ | -0.005 | 0.908 |  |  | 0.249 | 0.007 | ****** |
| daytime sleepiness ^b^ | 0.014 | 0.702 |  |  | -0.032 | 0.685 |  |
| **Sleep problems** |  |  |  |  |  |  |  |
| CSHQ Total Score ^a^ | 0.445 | <0.001 | ******* |  | 0.446 | <0.001 | ******* |
| bedtime resistance ^b^ | 0.070 | 0.126 |  |  | 0.064 | 0.511 |  |
| sleep onset delay ^b^ | 0.179 | <0.001 | ******* |  | 0.024 | 0.771 |  |
| sleep duration ^b^ | 0.245 | <0.001 | ******* |  | 0.212 | 0.008 | ****** |
| sleep anxiety ^b^ | 0.080 | 0.057 |  |  | 0.166 | 0.078 |  |
| night awakening ^b^ | 0.122 | 0.002 | ****** |  | -0.064 | 0.396 |  |
| Parasomnias ^b^ | 0.199 | <0.001 | ******* |  | 0.089 | 0.312 |  |
| sleep disordered breathing ^b^ | -0.030 | 0.433 |  |  | 0.308 | <0.001 | ******* |
| daytime sleepiness ^b^ | 0.004 | 0.910 |  |  | 0.034 | 0.658 |  |
| **Attention problems** |  |  |  |  |  |  |  |
| CSHQ Total Score ^a^ | 0.147 | 0.001 | ******* |  | 0.383 | <0.001 | ******* |
| bedtime resistance ^b^ | -0.041 | 0.445 |  |  | 0.086 | 0.387 |  |
| sleep onset delay ^b^ | 0.033 | 0.479 |  |  | -0.040 | 0.629 |  |
| sleep duration ^b^ | 0.104 | 0.028 | ***** |  | 0.094 | 0.247 |  |
| sleep anxiety ^b^ | 0.067 | 0.202 |  |  | 0.024 | 0.802 |  |
| night awakening ^b^ | -0.004 | 0.926 |  |  | -0.025 | 0.747 |  |
| Parasomnias ^b^ | 0.039 | 0.426 |  |  | 0.107 | 0.230 |  |
| sleep disordered breathing ^b^ | 0.055 | 0.229 |  |  | 0.368 | <0.001 | ******* |
| daytime sleepiness ^b^ | 0.033 | 0.456 |  |  | 0.124 | 0.118 |  |
| **Aggressive behavior** |  |  |  |  |  |  |  |
| CSHQ Total Score ^a^ | 0.233 | <0.001 | ******* |  | 0.308 | <0.001 | ******* |
| bedtime resistance ^b^ | 0.077 | 0.143 |  |  | 0.049 | 0.639 |  |
| sleep onset delay ^b^ | 0.033 | 0.464 |  |  | -0.036 | 0.681 |  |
| sleep duration ^b^ | 0.114 | 0.013 | ***** |  | 0.098 | 0.251 |  |
| sleep anxiety ^b^ | -0.049 | 0.340 |  |  | 0.038 | 0.702 |  |
| night awakening ^b^ | -0.012 | 0.788 |  |  | 0.018 | 0.821 |  |
| Parasomnias ^b^ | 0.148 | 0.002 | ****** |  | 0.095 | 0.310 |  |
| sleep disordered breathing ^b^ | 0.071 | 0.107 |  |  | 0.314 | 0.001 | ******* |
| daytime sleepiness ^b^ | 0.028 | 0.515 |  |  | 0.035 | 0.670 |  |
| **Stress** |  |  |  |  |  |  |  |
| CSHQ Total Score ^a^ | 0.163 | <0.001 | ******* |  | 0.358 | <0.001 | ******* |
| bedtime resistance ^b^ | -0.065 | 0.210 |  |  | -0.022 | 0.828 |  |
| sleep onset delay ^b^ | 0.045 | 0.303 |  |  | -0.015 | 0.860 |  |
| sleep duration ^b^ | 0.053 | 0.232 |  |  | 0.156 | 0.060 |  |
| sleep anxiety ^b^ | 0.056 | 0.262 |  |  | 0.096 | 0.325 |  |
| night awakening ^b^ | 0.022 | 0.625 |  |  | -0.028 | 0.725 |  |
| Parasomnias ^b^ | 0.092 | 0.051 |  |  | 0.106 | 0.242 |  |
| sleep disordered breathing ^b^ | 0.030 | 0.493 |  |  | 0.329 | <0.001 | ******* |
| daytime sleepiness ^b^ | 0.086 | 0.039 | ***** |  | 0.073 | 0.365 |  |

* 0.01＜p≤0.05；** 0.001＜p≤0.01；*** p≤0.001

^a^ Age of children and SRS total score were taken as predictive variables, and CSHQ total score was taken as independent variable.

^b^ Age of children, sex and SRS total score were taken as predictive variables, all CSHQ subscales were included in the regression equation.

Table S2. Linear regression analysis of parasomnias items on children behavior problems ^a^

|  | ASD | | |  | TD | | |
| --- | --- | --- | --- | --- | --- | --- | --- |
|  | β | p |  |  | β | p |  |
| **Emotionally reactive** |  |  |  |  |  |  |  |
| Wets the bed | 0.083 | 0.068 |  |  | 0.028 | 0.748 |  |
| Talks during sleep | 0.032 | 0.459 |  |  | 0.012 | 0.901 |  |
| Restless and moves a lot | 0.056 | 0.213 |  |  | 0.013 | 0.878 |  |
| Sleepwalks | -0.009 | 0.830 |  |  | -0.014 | 0.871 |  |
| Grinds teeth | 0.033 | 0.452 |  |  | 0.051 | 0.560 |  |
| Awakens screaming | 0.088 | 0.073 |  |  | 0.180 | 0.067 |  |
| Nightmares | 0.114 | 0.019 | ***** |  | 0.151 | 0.137 |  |
| **Anxious/depressed** |  |  |  |  |  |  |  |
| Wets the bed | 0.064 | 0.173 |  |  | 0.050 | 0.553 |  |
| Talks during sleep | 0.061 | 0.176 |  |  | 0.130 | 0.148 |  |
| Restless and moves a lot | 0.029 | 0.527 |  |  | -0.043 | 0.605 |  |
| Sleepwalks | 0.048 | 0.278 |  |  | 0.017 | 0.837 |  |
| Grinds teeth | 0.050 | 0.274 |  |  | 0.088 | 0.295 |  |
| Awakens screaming | 0.073 | 0.149 |  |  | 0.233 | 0.015 | ***** |
| Nightmares | 0.072 | 0.152 |  |  | 0.087 | 0.372 |  |
| **Somatic complaints** |  |  |  |  |  |  |  |
| Wets the bed | 0.144 | 0.002 | ****** |  | 0.032 | 0.717 |  |
| Talks during sleep | -0.003 | 0.949 |  |  | 0.091 | 0.326 |  |
| Restless and moves a lot | 0.107 | 0.021 | ***** |  | -0.076 | 0.372 |  |
| Sleepwalks | 0.095 | 0.032 | ***** |  | 0.028 | 0.747 |  |
| Grinds teeth | 0.091 | 0.044 | ***** |  | 0.070 | 0.422 |  |
| Awakens screaming | 0.149 | 0.004 | ****** |  | 0.160 | 0.102 |  |
| Nightmares | -0.010 | 0.845 |  |  | 0.136 | 0.178 |  |
| **Withdrawn** |  |  |  |  |  |  |  |
| Wets the bed | 0.098 | 0.014 | ***** |  | 0.009 | 0.919 |  |
| Talks during sleep | -0.009 | 0.815 |  |  | 0.111 | 0.227 |  |
| Restless and moves a lot | 0.034 | 0.388 |  |  | -0.003 | 0.967 |  |
| Sleepwalks | -0.060 | 0.114 |  |  | -0.011 | 0.897 |  |
| Grinds teeth | -0.004 | 0.911 |  |  | 0.060 | 0.484 |  |
| Awakens screaming | 0.017 | 0.702 |  |  | 0.122 | 0.210 |  |
| Nightmares | -0.023 | 0.590 |  |  | 0.194 | 0.055 |  |
| **Sleep problems** |  |  |  |  |  |  |  |
| Wets the bed | 0.107 | 0.010 | ****** |  | 0.017 | 0.847 |  |
| Talks during sleep | 0.101 | 0.012 | ***** |  | 0.067 | 0.459 |  |
| Restless and moves a lot | 0.174 | <0.001 | ******* |  | 0.030 | 0.717 |  |
| Sleepwalks | -0.029 | 0.469 |  |  | -0.012 | 0.893 |  |
| Grinds teeth | 0.016 | 0.686 |  |  | 0.029 | 0.733 |  |
| Awakens screaming | 0.192 | <0.001 | ******* |  | 0.268 | 0.006 | ****** |
| Nightmares | 0.162 | <0.001 | ******* |  | 0.104 | 0.296 |  |
| **Attention problems** |  |  |  |  |  |  |  |
| Wets the bed | 0.014 | 0.762 |  |  | 0.060 | 0.488 |  |
| Talks during sleep | -0.021 | 0.642 |  |  | 0.138 | 0.134 |  |
| Restless and moves a lot | 0.109 | 0.018 | ***** |  | -0.010 | 0.906 |  |
| Sleepwalks | -0.062 | 0.161 |  |  | 0.006 | 0.945 |  |
| Grinds teeth | 0.047 | 0.300 |  |  | 0.109 | 0.203 |  |
| Awakens screaming | 0.006 | 0.902 |  |  | 0.083 | 0.392 |  |
| Nightmares | 0.009 | 0.860 |  |  | 0.152 | 0.128 |  |
| **Aggressive behavior** |  |  |  |  |  |  |  |
| Wets the bed | 0.119 | 0.009 | ****** |  | 0.024 | 0.780 |  |
| Talks during sleep | 0.003 | 0.943 |  |  | 0.047 | 0.611 |  |
| Restless and moves a lot | 0.042 | 0.341 |  |  | -0.031 | 0.718 |  |
| Sleepwalks | -0.022 | 0.601 |  |  | 0.051 | 0.560 |  |
| Grinds teeth | 0.081 | 0.062 |  |  | 0.097 | 0.262 |  |
| Awakens screaming | 0.058 | 0.231 |  |  | 0.209 | 0.033 | ***** |
| Nightmares | 0.160 | 0.001 | ******* |  | 0.102 | 0.310 |  |
| **Stress** |  |  |  |  |  |  |  |
| Wets the bed | 0.036 | 0.410 |  |  | 0.008 | 0.931 |  |
| Talks during sleep | 0.000 | 0.992 |  |  | 0.096 | 0.296 |  |
| Restless and moves a lot | 0.082 | 0.059 |  |  | 0.055 | 0.518 |  |
| Sleepwalks | 0.003 | 0.947 |  |  | 0.015 | 0.859 |  |
| Grinds teeth | 0.003 | 0.947 |  |  | 0.056 | 0.519 |  |
| Awakens screaming | 0.078 | 0.101 |  |  | 0.191 | 0.051 |  |
| Nightmares | 0.096 | 0.043 | ***** |  | 0.092 | 0.357 |  |

* 0.01＜p≤0.05；** 0.001＜p≤0.01；*** p≤0.001

^a^ Age of children, sex and SRS total score were taken as predictive variables, all parasomnias items were included in the regression equation

Table S3. Frequency of parasomnias and sleep disordered breathing problems in two groups ^a^.

|  | ASD (%) | |  | TD (%) | | p | | | |
| --- | --- | --- | --- | --- | --- | --- | --- | --- | --- |
|  | M | F |  | M | F | ASD | TD | M | F |
|  |  |  |  |  |  | M VS F | M VS F | ASD VS TD | ASD VS TD |
| **parasomnias** |  |  |  |  |  |  |  |  |  |
| Wets the bed | 24.3 | 40.3 |  | 14.9 | 15.1 | 0.009** | 1.000 | 0.049* | 0.001*** |
| Talks during sleep | 4.0 | 4.2 |  | 11.2 | 17.4 | 1.000 | 0.297 | 0.007** | 0.011* |
| Restless and moves a lot | 39.8 | 52.8 |  | 31.8 | 32.6 | 0.051 | 1.000 | 0.146 | 0.015* |
| Sleepwalks | 2.0 | 1.4 |  | 9.3 | 4.7 | 1.00 | 0.270 | 0.001*** | 0.277 |
| Grinds teeth | 28.3 | 11.1 |  | 26.2 | 16.3 | 0.001*** | 0.115 | 0.716 | 0.360 |
| Awakens screaming | 20.1 | 26.4 |  | 7.5 | 4.7 | 0.273 | 0.553 | 0.001*** | < 0.001*** |
| Nightmares | 10.5 | 16.6 |  | 5.6 | 4.7 | 0.162 | 1.000 | 0.074 | 0.017* |
| **sleep disordered breathing** |  |  |  |  |  |  |  |  |  |
| Snores loudly | 26.1 | 20.8 |  | 18.7 | 11.6 | 0.379 | 0.231 | 0.129 | 0.120 |
| Stops breathing | 2.8 | 2.8 |  | 2.8 | 3.5 | 1.000 | 1.000 | 0.646 | 1.000 |
| Snorts and gaps | 6.3 | 6.9 |  | 2.8 | 1.2 | 0.797 | 0.630 | 0.234 | 0.093 |

* 0.01＜p≤0.05；** 0.001＜p≤0.01；*** p≤0.001

^a^ Assessed by parents as “sometimes” or “usually” were identified as having specific sleep disturbance.

Abbreviations: M, male; F, female.

Table S4. Linear regression analysis of sleep disordered breathing items on children behavioral problems ^a^

|  | ASD | | |  | TD | | |
| --- | --- | --- | --- | --- | --- | --- | --- |
|  | β | p |  |  | β | p |  |
| **Emotionally reactive** |  |  |  |  |  |  |  |
| Snores loudly | 0.049 | 0.283 |  |  | 0.059 | 0.499 |  |
| Stops breathing | 0.027 | 0.623 |  |  | 0.180 | 0.096 |  |
| Snorts and gaps | 0.041 | 0.443 |  |  | 0.160 | 0.131 |  |
| **Anxious/depressed** |  |  |  |  |  |  |  |
| Snores loudly | 0.084 | 0.071 |  |  | 0.075 | 0.378 |  |
| Stops breathing | 0.032 | 0.565 |  |  | 0.185 | 0.079 |  |
| Snorts and gaps | 0.034 | 0.536 |  |  | 0.209 | 0.043 | ***** |
| **Somatic complaints** |  |  |  |  |  |  |  |
| Snores loudly | 0.031 | 0.513 |  |  | 0.241 | 0.043 | ***** |
| Stops breathing | -0.029 | 0.612 |  |  | 0.362 | 0.045 | ***** |
| Snorts and gaps | 0.093 | 0.103 |  |  | 0.363 | 0.218 |  |
| **Withdrawn** |  |  |  |  |  |  |  |
| Snores loudly | -0.022 | 0.582 |  |  | 0.069 | 0.422 |  |
| Stops breathing | 0.025 | 0.594 |  |  | 0.241 | 0.024 | ***** |
| Snorts and gaps | 0.011 | 0.816 |  |  | 0.148 | 0.155 |  |
| **Sleep problems** |  |  |  |  |  |  |  |
| Snores loudly | 0.112 | 0.014 | ***** |  | 0.160 | 0.061 |  |
| Stops breathing | -0.051 | 0.350 |  |  | 0.269 | 0.047 | ***** |
| Snorts and gaps | 0.087 | 0.106 |  |  | 0.139 | 0.175 |  |
| **Attention problems** |  |  |  |  |  |  |  |
| Snores loudly | 0.069 | 0.129 |  |  | 0.194 | 0.020 | ***** |
| Stops breathing | 0.002 | 0.964 |  |  | 0.214 | 0.037 | ***** |
| Snorts and gaps | 0.021 | 0.706 |  |  | 0.143 | 0.156 |  |
| **Aggressive behavior** |  |  |  |  |  |  |  |
| Snores loudly | 0.051 | 0.261 |  |  | 0.063 | 0.453 |  |
| Stops breathing | 0.025 | 0.650 |  |  | 0.232 | 0.026 | ***** |
| Snorts and gaps | 0.090 | 0.095 |  |  | 0.215 | 0.035 | ***** |
| **Stress** |  |  |  |  |  |  |  |
| Snores loudly | 0.070 | 0.112 |  |  | 0.163 | 0.055 |  |
| Stops breathing | 0.034 | 0.521 |  |  | 0.194 | 0.063 |  |
| Snorts and gaps | 0.054 | 0.298 |  |  | 0.164 | 0.167 |  |

* 0.01＜p≤0.05；** 0.001＜p≤0.01；*** p≤0.001

^a^ Age of children, sex and SRS total score were taken as predictive variables, all parasomnias items were included in the regression equation
